# Searching for New Human Behavior Model in the Climate Change Age: Analyzing the Impact of Risk Perception and Government Factors on Intention–Action Consistency in Particulate Matter Mitigation

**DOI:** 10.3390/ijerph191711068

**Published:** 2022-09-04

**Authors:** Yeji Shin, Seoyong Kim, Sohee Kim

**Affiliations:** 1 Energy Transformation Policy Research Center, Social Science Research Institute, Ajou University, Suwon 16499, Korea; 2 Department of Public Administration, Ajou University, Suwon 16499, Korea

**Keywords:** particulate matter, government factors, intention–action consistency, risk perception paradigm

## Abstract

This study aims to analyze factors influencing citizens’ intentions to take protective action against particulate matter (PM) and their actual actions in response to PM. There were few research on the role of government factors and the issue of intention–action inconsistency in the context of PM mitigation action. Therefore, this study set not only variables in the risk perception paradigm but also ones in government factors as independent variables, while intention and action in response to PM were set as dependent variables. This study’s analysis was based on survey data collected from Korean people. For representativeness of the samples, this study adopted the quota sampling method, considering region, gender, and age. Five hundred respondents finished the survey. To verify the hypotheses, this study used regression and binomial logistic analysis. Analysis showed that (1) negative emotions, trust, knowledge, government competency, policy satisfaction, and policy awareness had significant effects on intention and action in response to PM, and (2) perceived benefits only affected intention, whereas government accountability only affected action. Logistic analysis showed that there were groups in which intentions and actions did not match. Negative emotions and government competence induce intention–action consistency, whereas the perceived benefits and trust in government tend to encourage inconsistency. Knowledge is a variable that induces both consistency and inconsistency in the intention–action relationship. The determinant structures of independent variables affecting the likelihood of belonging to the four groups differed.

## 1. Introduction

**Figure 1:**
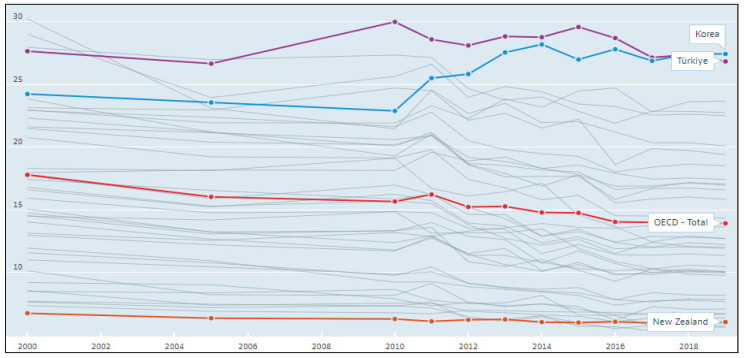
Air pollution exposure, expressed as exposure to PM 2.5 in micrograms per cubic meter for 2000–2019. Source: OECD, Air pollution exposure (indicator) [1].

Particulate matter (PM) is a critical health hazard. PM negatively affects the respiratory system and causes related diseases. When looking at statistics, PM risk in Korea is at a very serious level compared with other countries. According to the OECD [1], as shown in Figure 1, among OECD countries, Korea has the highest exposure to PM 2.5. In 2019, the exposure to PM 2.5 in New Zealand was 6.05 µg/m^3^ (micrograms per cubic meter), while the OECD average was 13.93 µg/m^3^. In Korea, it was 27.45 µg/m^3^, or 4.54 times that of New Zealand and 2.30 times the OECD average. Furthermore, Figure 1 shows that the PM has been decreasing in many OECD countries during last 20 years. However, in Korea, it increased from 24.25 µg/m^3^ in 2000 to 27.45 µg/m^3^ in 2019 [1]. The levels of economic development and PM generally have an inverse relationship. As Korea has achieved significant economic development over the past few years, it was expected that PMs would decrease. However, in the case of Korea, PM has not decreased despite its economic development.

According to the statistics of the Health Insurance Review and Assessment Service in South Korea, the number of acute bronchitis patients directly affected by fine dust increased to 14.87 million in 2013, 15.11 million in 2014, 1.51 million in 2015, 15.81 million in 2016, and 16.22 million in 2017. In particular, as of 2017, Gyeonggi (4.15 million), Seoul (3.2 million), and Incheon (940,000) accounted for half of all patients in the metropolitan area [2].

Today, the participation of citizens is essential for the effective management of environmental hazards threatening public health. When discussing governance to solve environmental problems, it is common to consider not only the relationship between the government and business but also one between government, business, and civil society. Mitigation measures cannot be effectively implemented solely by the government without public cooperation. In tackling PM, individuals’ reduction efforts are as important as national public policy because they are not only victims of PM but also its producers. As the need for an active response from citizens for the effective implementation of policies has increased, response intention and action (i.e., response behavior) to PM has become an important research topic [3]. However, the existing literature reveals some theoretical limits, and they are as follows.

First, previous studies on PM did not consider inconsistency between intention and action. A lot of theoretical studies on topics other than the PM field have focused on intention–action inconsistency. After Sheeran and Webb [4] conducted a meta-analysis of 422 studies on intention and behavior, they found that intention provides a significant amount of predictive power for behavior and that the intention–behavior gap cannot be overlooked, which raises the need for management. Furthermore, since previous research tends to focus more on intention than action, they assumed that intention always precedes and directly influences action [5]. Moreover, there is an inconsistency and gap between intentions and actions, as the former is not always linked to the latter [6]. In order to solve the PM problem, the relationship between behavioral intention and actual behavior is particularly important. Since modifying behavior to curb PM entails costs and risks, intentions may not always be followed up with actions. Research over securing the consistent linkage between intention and action could provide clues for solving the PM problem.

Second, when exploring factors influencing the intention and response behavior toward PM, previous studies focus on individual psychological factors and risk communication factors. Although the psychological and communication factors influence people’s behaviors at the individual level, the government factor has played an important role in influencing the public at the policy level. However, although the government usually makes various efforts to respond to PM-related problems, previous studies tended to disregard the process of determining whether the government makes PM management or policy efforts for citizens’ safety. In relation to the PM issue, the theoretical focus on government factors such as the competence, trust, and satisfaction with government policies, which is the main management entity, has not been fully considered.

Therefore, the purpose of this study is to analyze the factors determining the response intention and response action to reduce PM. In particular, we explore the inconsistency between intention and action. To this end, we comprehensively examined the relationships between intention and action by dividing the respondents into four groups according to a two-by-two matrix. Moreover, this study attempts to focus on the role of government factors. To this end, we compare the explanatory power of government variables—government competence, trust, government accountability, policy satisfaction, and policy awareness—with variables from the psychometric paradigm (also called the risk perception paradigm). This study used data collected in the 2022 National Emission Air Quality National Consciousness Survey (*N* = 500).

## 2. Theory and Hypotheses

### 2.1. Factors Influencing PM Response Intentions and Actions

Response intention and action (i.e., response behavior) can be defined as individuals’ psychological will and behavioral responses to external stimuli. A wide variety of theories, such as the theory of reasoned action [7,8], the theory of planned behavior [5,9], Triandis’s [10] attitude–behavior theory, and protection motivation theory [11], have focused on intention because intention is believed to be the most immediate proximal important cause of behavior. These theories assume that people do what they intend to do. Because behavior and intention are very closely connected, the concept of behavioral intention was usually used. Behavioral intentions are instructions that people give to themselves to behave in certain ways [10] (p. 203). They are types of motivations and decisions that include both the direction (doing X vs. not doing X) and the intensity (i.e., how much time and effort to spend on the action) [6].

Many studies have shown how accurately intentions predict behaviors such as consumer and leisure decisions (e.g., Warshaw and Davis [12]), smoking [13], academic activities and achievements (e.g., Manstead and van Eekelen [14]), gambling (e.g., [15]), voting (e.g., [16]), occupational choices [17], and donating blood (e.g., [18]).

Previous studies on intention and behavior show that one of the main theoretical issues is the gap between intention and action [6]. Not all intentions are expressed in actions. The intention–action gap, which is a byproduct of the inconsistency between intention and action, is one of the classic theoretical topics in the social sciences. The theory of planned behavior (TPB), one of the widely used theories to study intent and behavior, assumes that the most important factor in determining “action” is “intention” [5,19]. Ajzen [5], who devised the theory of planned behavior, acknowledged that not all intentions lead to action and that intentions are frequently abandoned, or some are modified to respond to the situation. After conducting a meta-analysis of studies related to the theory of reasoned action and planned behavior theory, Sutton [20] pointed out that the reviewed theoretical models can explain approximately 40–50% of intention on average, but the explanatory power is decreased to 19–38% when it comes to following up intention with the intended behavior. Moreover, Sheeran [6] showed that there are two groups which do not show consistency between intention and behavior: inclined abstainers, who have positive intention but do not act, and disinclined actors, who have negative intention but do act. He also showed that among the two groups, inclined abstainers are more numerous than disinclined actors. After conducting a meta-analysis of 422 studies on the intention–action relationship, Sheeran and Webb [4] reported that although intention holds significant predictive explanation power for action, it is still important to study the gap between intention and action to help increase intention and action consistency. Although many studies have identified the influential power of intention, Lee et al. [21] reported that there is always an intention and action mismatch group because inconsistency between intention and behavior may usually occur.

Empirical studies have tried to find variables that can explain the gap between intention and action. For example, Sheeran [6] found that when looking only at the relationship between intention and behavior, what contributes to predicting behavior are issues of control, intention versus expectation, implementation intention, and cognitive and personality variables. However, since these variables refer to only intention and behavior themselves, they overlook the factors affecting them. Accordingly, many studies have tried to find variables that affect either intention or behavior and the gap between them.

Several exploratory studies focus on proximal and remote factors affecting intention and behavior rather than focusing on intention and behavior themselves. For example, based on the protection motivation theory, Orbell and Sheeran [22] revealed perceived susceptibility, perceived severity, worry, response efficacy, and self-efficacy as influencing factors. Moreover, based on a survey in the Swiss electricity market, Litvine and Wüstenhagen [23] showed that trust moderates the relationships between attitude and intention. After analyzing attitudinal inconsistency toward organic food in relation to purchasing intention and behavior, Tung et al. [24] reported that benefit certainty plays a role in narrowing the gap between willingness to pay (WTP) and behavior. Based on a meta-review of 58 articles, ElHaffar et al. [25] showed that not only intrapsychic factors (e.g., personal norms, perceived efficacy, green perception, inconsistent attitude, reason, justification, social awareness, habits, environmental consciousness, denial, conflicting goals, tokenism, perceived compromises, perceived utility, mental representation, and distance) but also contextual factors (e.g., price, social context, physical context, functional risk, temporal risk, availability, quality, communication, green trade-offs, social identity, reputation for the consumer, income, education, incentive, emotion, interaction, stimulation, and information) influence the gap between intention and behavior.

### 2.2. Intention–Action Consistency: Predictors of Intention and Behavior in PM Issues

In the field of PM, a few studies focus on which causal factors influence intention and action. Previous studies have shown several research trends.

First, previous studies examined intention and action separately rather than comparing them. For example, regarding the intention and action in PM mitigation, Park and Kim [26] distinguished between private and public trust to verify whether trust in government agencies affects PM response behavior. However, that study did not consider the role of intention.

Second, in terms of theoretical frameworks, as causal factors influencing the intention and action toward PM, previous studies adopted the psychometric paradigm, known as the risk perception paradigm [3,27]. This paradigm was proposed by Slovic et al. [28]. It assumes that the assessment of risk is not objective but based on individual subjective judgment. It stressed perception factors such as perceived risk, perceived benefit, trust, subjective knowledge, and image. Regarding PM, there have been a number of studies that focused on risk perception, as it is a hazardous factor. For example, mainly based on the psychometric paradigm, Jung and Kim [27] showed how not only self-efficacy, personal norms, information source credibility, psychological distance, and commitment to action but also perceived risk, perceived benefit, trust, and knowledge influence the judgement and action toward PM. In addition, Kim et al. [3] showed that risk perception, communication, and attribution significantly influence the person or public mitigation and adaptation. In particular, perceived benefit, perceived risk, negative emotion, the receiver’s ability, and external attribution have an impact on them. These existing studies mainly focused on the individual response to fine dust as the dependent variable.

Third, there are emerging studies focusing on the role of government in PM policy and management. Kwon [29] showed that local governments compete for fine dust management policies. Park and Lee [30] showed the utilization of policy measures to reduce fine dust. Kim et al. [31] estimated the sociocultural willingness to pay to resolve fine dust problems. Kim and Lee [32] analyzed the failure factors of PM policies. However, since these studies focused on only a part of the role of the government and policies, they failed to systematically analyze the roles of the various functions performed by them. Therefore, this study intends to explore various functions of government and policy in response to PM.

Based on these previous studies, we suggest the research model shown in Figure 1. This study aims to identify the predictors of intention and action. It also examines the factors influencing a group’s affiliations based on (in)consistency between intention and action. To analyze these affiliations, based on the respondents’ answers to the questions to measure the intention and action response to PM, this study constructs four groups: (1) a low-intention low-action group, (2) a low-intention high-action group, (3) a high-intention low-action group, and (4) a high-intention high-action group. Comparative studies on intention and action are needed because they are distinctive theoretical concepts between which inconsistency likely often exists.

**Figure 2:**
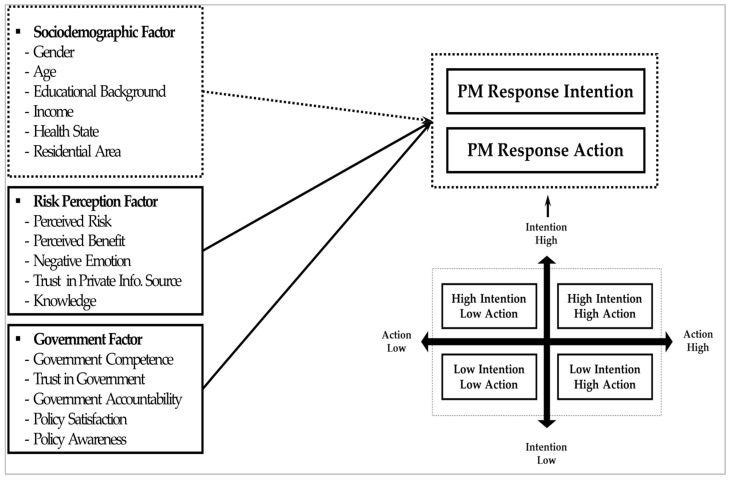
Research model.

As shown in Figure 2, as the predictors, we set up two independent factors: The risk perception factor and government factor. Because PM is a dangerous substance, people’s perception plays a decisive role in intention and behavior. We examined the role of perception variables such as perceived risk, perceived benefit, negative emotion, knowledge, and trust. Moreover, since the government as an official actor promotes many policies to lower PM levels, it is important to examine how those policies influence public intention and behavior. Therefore, we focused on government factors, namely government competence, trust in government, government accountability, policy satisfaction, and policy awareness. Finally, we set demographic factors (gender, age, educational background, income, health state, and residential area) as control variables. The theories and hypotheses presented in the research model are reviewed in the next chapter.

### 2.3. Risk Perception Factors and Hypotheses

#### 2.3.1. Perceived Risk

Perceived risk refers to an individual’s subjective assessment of any risk [28]. In other words, it is the degree of the sense of danger [33]. 

Ko and Song [34] demonstrated that not only anxiety but also perceived risk have a significant effect on the intention to take preventive action against fine dust. Additionally, after analyzing Chinese citizens’ actions against PM, Yang and Jo [35] showed that the level of PM prevention behavior was high when the perceived risk and self-efficacy were high. Park and Kim [26] showed that perceived risk has a positive effect on response behavior against fine dust. Kim et al. [3] showed that higher perceived risk for PM increases personal mitigation behavior. Those studies suggested the following hypotheses:

**Hypothesis** **1-1** **(H1-1).** *Perceived risks will have a positive effect on the intention to respond to PM.*

**Hypothesis** **1-2** **(H1-2).** *Perceived risks will have a positive effect on action in response to PM.*

#### 2.3.2. Perceived Benefit

The variable of perceived benefit is the opposite of perceived risk [36]. It can be defined as an individual’s perception of utility from material or non-material rewards [26]. There are very few studies verifying the relationship between the benefits of PM, response intention, and behavior because, in general, there is no direct benefit from PM, only damage. Park and Kim [26] examined the policy benefits of resolving the PM issue and not PM itself. When people expected a higher benefit, they took more response actions against PM. Jung and Kim [27] suggested that perceived benefits have a positive effect on trust in rumors about PM; the higher the benefit from solving the PM problems, the more likely they are to take the risk of PM seriously. Kim et al. [3] demonstrated that higher perception of benefits from solving the PM problem increases personal adaptation and mitigation behavior. The following hypotheses can be derived from previous studies:

**Hypothesis** **2-1** **(H2-1).** *Perceived benefits will have a positive effect on the intention to respond to PM.*

**Hypothesis** **2-2** **(H2-2).** *Perceived benefits will have a positive effect on action in response to PM.*

#### 2.3.3. Negative Emotion

Although negative emotions are unpleasant states of mind, they can be useful when they help encourage healthy behavior [37,38]. Jang and Cho [39] showed that the more negative feelings restaurant managers had about PM, the higher their precautionary behavior against it was. Cha and Cho [38] confirmed that among domestic Internet users, negative emotions have a positive influence on the intention to prevent PM. Yang and Cho [35] analyzed the effect of anxiety, i.e., a negative emotion, and they showed that the higher the anxiety, the higher the response behavior to PM. In addition, they demonstrated that compared with other factors, affective factors play an important role in overwhelming risk perception factors. Negative emotion increased not only personal mitigation and adaptation but also public adaptation [3]. The above studies led us to the following hypotheses:

**Hypothesis** **3-1** **(H3-1).** *Negative emotions will have a positive effect on the intention to respond to PM* *.*

**Hypothesis** **3-2** **(H3-2).** *Negative emotions will have a positive effect on action in response to PM* *.*

#### 2.3.4. Trust in Private Information Sources

Trust is one of the key factors in risk management, as a lack of trust can lead to policy failure [40]. After Hong and Lee [41] classified information about PM into official and informal information, they put trust in public sources in the former category and trust in private sources in the latter category. They showed that the higher the level of trust in public information, the higher the intention to act against PM. Based on measurement of trust in the World Meteorological Organization and aliased environmental organizations that convey information on fine dust, Kang and Kim [42] revealed that the interactions between the source credibility and attribution frames have a significant effect on the intention to prevent PM. From the perspective of the risk perception paradigm, trust generally refers to trust in the government or public institutions, which are the main actors in risk management [40,43,44]. In the case of PM, not only public institutions but also private institutions play an important role. Moreover, Kim et al. [3] showed that a source’s credibility affects personal adaptation. This study assumes that trust in private actors affects intentions and behaviors:

**Hypothesis** **4-1** **(H4-1).** *Trust in private information sources will have a positive effect on the intention to respond to PM* *.*

**Hypothesis** **4-2** **(H4-2).** *Trust in private information sources will have a positive effect on action in response to PM* *.*

#### 2.3.5. Knowledge

Knowledge can be understood as the amount of subjectively perceived or objective information about a particular object. Kwon and Kim [45] analyzed the fine dust response behavior of mothers and found that the higher the knowledge of PM they had, the higher the level of response behavior they showed. Meanwhile, Park and Kim [26] hypothesized that the higher the level of knowledge related to PM, the higher the level of response behavior. But regression analysis did not support the hypothesis.

Weng et al. [46] found no difference in PM2.5 knowledge, PM2.5 preventive attitudes, or PM2.5 preventive behavioral intentions among elderly individuals with or without chronic diseases. Additionally, according to Kim et al. [3], knowledge has no impact on personal adaptation behavior against PM:

**Hypothesis** **5-1** **(H5-1).** *Knowledge will have a positive effect on the intention to respond to PM* *.*

**Hypothesis** **5-2** **(H5-2).** *Knowledge will have a positive effect on action in response to PM* *.*

### 2.4. Government Factors

#### 2.4.1. Government Competence

Government competence means the capability of the government to manage the environment and solve social problems [47]. Previous studies have verified the relationship between government competence and intention and action against PM. Yoon and Kim [48] confirmed a positive relationship between government competency and perceived social safety. After analyzing the effect of technology perception on its acceptance, Jeong et al. [49] showed that the government’s technology competence has a significant moderating effect on satisfaction with e-governance. Thus, even if new technologies generate negative side effects, the public will worry less about them if the government has the competence to properly control and manage them. Kim [50] found that the higher the perception of government management competence, the lower the perception of risk. However, this relationship was not statistically significant. Based on these findings, the following hypotheses were established:

**Hypothesis** **6-1** **(H6-1).** *Government competence will have a positive effect on the intention to respond to PM* *.*

**Hypothesis** **6-2** **(H6-2).** *Government competence will have a positive effect on action in response to PM* *.*

#### 2.4.2. Trust in Government

Although there are differences in the conceptual definition of trust in government among scholars, it can be regarded as a kind of positive attitude held by citizens toward the government [51]. According to Kim et al. [3], trust in government increases personal adaptation and public mitigation in response to PM. Kim and Moon [52] pointed out that if the level of trust in government is high, individuals will show passive action in disaster prevention because they strongly believe that the government will protect them. Overall, trust in the government is expected to have a positive effect on intention and behavior, and thus the following hypotheses were established:

**Hypothesis** **7-1** **(H7-1).** *Trust in government will have a positive effect on the intention to respond to PM* *.*

**Hypothesis** **7-2** **(H7-2).** *Trust in government will have a positive effect on action in response to PM* *.*

#### 2.4.3. Government Accountability

Government accountability means that the government acts with an obligation toward its sovereign citizens. In particular, government accountability concerns the issue of attribution of responsibility for negative events. Therefore, the role of government accountability is based on the attribution theory that specifies who the accountability and blame are transferred toward when a dangerous situation occurs [53]. Kang and Kim [42] conducted a study to examine whether there is a change in response behavior depending on the attribution frame. They showed that when PM issues become serious problems, the precautionary action and participation intention occur more in external attribution to China than in internal attribution to domestic actors. The attribution can be applied to the relationships between individuals and the government. When the accountability of blame is attributed to the government, individuals will show less intention and action toward the target goals. Kim [33] showed that when individuals blame the government rather than themselves for disastrous events such as COVID-19, they reveal very low trust in the government. This suggests that more attribution to the government can lower individuals’ response behaviors against negative situations. The following hypotheses were established:

**Hypothesis** **8-1** **(H8-1).** *Government accountability will have a negative effect on the intention to respond to PM* *.*

**Hypothesis** **8-2** **(H8-2).** *Government accountability will have a negative effect on action in response to PM* *.*

#### 2.4.4. Policy Satisfaction

Satisfaction refers to the degree of utility obtained from a specific target. Satisfaction with the government is a kind of positive evaluation that individuals feel toward it and its policies. A policy is like a service product provided by the government; the higher its quality, the higher the satisfaction with it. Previous studies show that satisfaction with policies or related institutions encourages citizens to take action. For example, Yoo et al. [54] showed the structural relationship between consumer policy awareness, policy satisfaction, and response behaviors. Lee et al. [55] showed that higher customer satisfaction with the green certification system had a positive influence on the behavioral intention toward it. Kim [56] found that in the COVID-19 situation, the action of social distancing had a positive correlation and causal relationship with satisfaction with the K-prevention policy and response from government agencies. Based on these findings, we provide the following hypotheses:

**Hypothesis** **9-1** **(H9-1).** *Policy satisfaction will have a positive effect on the intention to respond to PM* *.*

**Hypothesis** **9-2** **(H9-2).** *Policy satisfaction will have a positive effect on the action in response to PM* *.*

#### 2.4.5. Policy Awareness

A certain knowledge and awareness of the policy induces positive cooperative functions in the actors, which induces support for the policy and related actions. Yoo et al. [54] showed that the higher the consumer’s awareness of food safety regulation policies, the higher the response behavior to food safety. In addition, Lee et al. [55] showed that policy awareness about green certification had a significant structural relationship with the perception of service quality and behavioral intention, which are mediated by customer satisfaction. Park and Jeon [57] demonstrated the significant positive impact of policy awareness on the intention to participate in the project:

**Hypothesis** **10-1** **(H10-1).** *Policy awareness will have a positive effect on the intention to respond to PM* *.*

**Hypothesis** **10-2** **(H10-2).** *Policy awareness will have a positive effect on the action in response to PM* *.*

## 3. Sample and Measures

This study used data collected by the research project “2022 National Discharge Air Quality People’s Consciousness Survey,” which is financially supported by the Fine Dust Information Center. Data collection was conducted by Korea Research, a polling company, from 21 February to 27 February 2022 through the Internet survey method. The survey respondents were 19 years or older and of Korean nationality. For representativeness of the samples, this study adopted the quota sampling method, considering region, gender, and age. Finally, 500 respondents finished the survey. To verify the hypotheses, this study used SPSS 22.0, a statistical package provided by IBM.

Among the 500 respondents, 246 were men (50.8%), and 254 were women (49.2%). Regarding age groups, 150 respondents (30.0%) were in their 60s or older, followed by 99 in their 50s (19.8%), 92 in their 40s (18.4%), 83 in their 20s (16.6%), and 76 in their 30s (15.2%). Regarding the academic background, 334 (66.8%) were enrolled in college or graduated from college, and the other 166 (33.4%) were high school graduates or lower. Regarding the residential area, 285 (57.0%) lived in large cities, followed by 187 (37.4%) in small- and medium-sized cities and 28 (5.6%) in rural areas. In Table 1, we compared the sample to the population by using statistics from a census conducted by the Korean government in 2020. The following table shows that the proportions by gender and age in the population and sample were almost similar.

Most of the measurement questions for analysis consisted of asking the respondent the degree of consent (please tell me how much you agree with the following statement), and the response was generally measured on a 5-point Likert scale (1 = do not agree at all, 2 = disagree, 3 = neutral, 4 = agree, and 5 = strongly agree). We used questions which were validated by Kim et al. [58] and Wang and Kim [59]. We added this explanation to the text. Trust in private and public sources of information was measured by asking the following question: “How much do you trust the information from the sources below?” The degree of trust was measured by using a 5-point scale (1 = very distrustful, 2 = slightly distrustful, 3 = neutral, 4 = slightly trust, and 5 = trust very much). Policy awareness was measured by asking the following question: “Are you aware of the following policies?” This was answered on a 5-point scale (1 = do not know not at all, 2 = do not know, 3 = know a little bit, 4 = I know, and 5 = know very well).

When a reliability test for measurements was conducted using Cronbach’s α, all Cronbach’s α values were found to be 0.61 or higher. The measurement items and reliability test results are shown in Table 2 below. In order to examine the validity of the measurement, a factor analysis was performed (please see Appendix A and Appendix B).

For intention and behavior, a post hoc analysis was performed by using the Tukey, Scheffe, and Bonferroni tests. In terms of intention, there are systematical differences between those in their 20s and 60s, 30s and 40s, and 50s and 60s in age, between high school graduates and those with a graduate degree or higher in the case of the education level, and between living in large cities and rural areas in the case of cities. In terms of behavior, there was a difference between those in their 20s, 40s, 50s, and 60s, those in their 30s, 40s, 50s, and 60s, and those in their 40s and 50s. In terms of education, there was no difference between groups. In the case of residence, there was a difference in time between the large cities and small and medium cities.

## 4. Analysis and Findings

### 4.1. Descriptive Analysis

**Figure 3:**
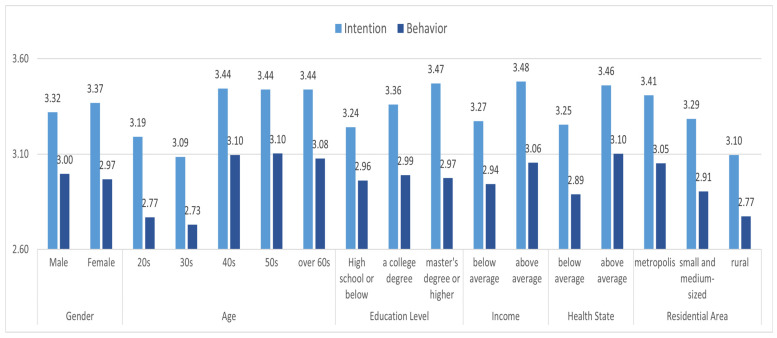
Mean difference analysis results for sociodemographic factors.

Before exploring the determinants of response intention and behavior against PM, we checked whether there was a significant difference in the mean in intention and action for each focused variable. For the calculation of these variables, the mean value was used when there were multiple measurement items. To know the difference between groups, an independent sample *t*-test was performed in the case of two groups, and a one-way variance test was performed for three groups. Figure 3 shows the result of analyzing the mean difference in intention and action against PM according to sociodemographic factors.

In the case of gender, there was no significant difference in intention and action between men and women, but in the case of age, there was a statistically significant difference in both response intention and behavior across the five age groups. Interestingly, the behavioral intention was higher in women, but the behavior was higher in men.

When observing the difference according to age, the F statistic was 6.366, showing a significant difference in the case of response intention at a significance level of 0.001. The level of intention was highest among those in their 60s or older (3.34), followed by 50s > 40s > 20s > 30s. In the case of response action, the F statistic value was 6.353, showing a statistical significance level of 0.001. The level of action was in the order of 50s > 40s > 60s > 20s > 30s. Both intentions and action against PM were higher among those in their 40s or older than in younger respondents in their 20s or 30s. This implies that older people are more resourceful, which drives both intentions and behaviors.

Regarding education, there was a significant difference only in intention and not action. In the case of education and intention, the F statistic value was 2.580, and the significance probability was 0.077. The level of intention was in the order of those who graduated from university or higher > college graduate > high school graduate and below. These results suggest that the accumulation of knowledge through formal education increases the intention to respond to PM.

There was a significant difference in income level. The level of intention was higher in groups with above-average incomes, and the *t* statistic was −3.457 with a significance probability of 0.001. On the other hand, the level of response behavior was statistically significantly higher in the high-income group than the low-income group.

In the case of subjective health, it was composed of continuous variables, so it was classified into two groups based on the average value. Those with bad health showed a higher level of intention than the healthy group. The *t* statistic was –3.603, and the probability of significance was 0.000. Such patterns appeared again in the case of behaviors. The former group showed more response than the latter group. (The *t* statistic was 3.269, and the significance probability was 0.001.) Since fine dust is a first-class carcinogen, as declared by the World Health Organization, it causes various diseases. It was assumed that the unhealthy group would respond more to fine dust. However, the result was the opposite. It seems that good health acts as a factor that motivates individuals to actively respond to external health threat factors.

Regarding the residential environment, the level of intention for action and behavior was high in the order of large cities > small- and medium-sized cities > rural areas. (The F statistics were 4.312, the significance probability was 0.014 in the case of intention, the F statistic value was 3.681, and the significance probability was 0.026 in case of behavior) The higher concentration of vehicles and lesser forest coverage in large cities leads to higher levels of fine dust.

### 4.2. Correlation Analysis

Table 3 below shows the results of correlation analysis between variables in the risk perception paradigm, the government factor, and intention and behavior.

The last two rows in Table 3 show that perceived risk had a significant positive correlation with intention and action (0.264 and 0.168 coefficients, respectively). Perceived risk had a higher association with intention than with behavior. In general, actions are more difficult than intentions because they require an actual cost input.

Perceived benefits had positive correlations with response intentions and behaviors against PM. As with perceived risk, the coefficient value was larger for intention than for action. Compared with perceived risk, the difference in the correlation coefficient values between intention and behavior in perceived benefit was larger. These results suggest that perceived benefit may be a powerful factor causing inconsistency between intention and behavior.

Negative emotions had positive correlations with response intentions and response behaviors against PM. The correlation coefficient value of negative emotions was larger than that of perceived benefits and perceived risks based on rational calculations. These results indicate that emotional factors are more influential than non-emotional factors in intentions and actions against fine dust.

Trust in private sources of information showed a positive correlation with response intentions and response behaviors against PM. This suggests that online information that individuals access every day has a positive effect on fine dust-related intentions and behaviors. Although it is not known how the specific nature of such information (that is, whether it is positive or negative) affects intentions and behaviors, our findings suggest that trust is important.

Knowledge showed a positive correlation with PM-related response intention and response behavior. There are two interesting points here. First, among the four variables analyzed, knowledge had the largest correlation coefficients. Thus, knowledge exerts a great influence on intention and behavior. Second, among the other three variables, the coefficient of intention was larger than that of action, indicating that knowledge is a key driving factor in converting intention into action.

Among the five variables of the risk perception paradigm, response intention had the greatest relationship with knowledge, followed by negative emotion, trust, perceived benefit, and perceived risk. Response behavior had a similar pattern of relationships. However, contrasted with intention, behavior was associated with perceived risk rather than perceived benefit.

Examining the relationship between the five variables in government factors and behavioral intention and action revealed the following. First, government competence has a positive relationship with intention and action. The small difference between the coefficient values of intention and action indicates that belief in government competence has an equal influence on both intentions and actions.

Second, trust in government has a positive relationship with intention and action, and the correlation with intentions is higher. Notably, the coefficient value was small compared with that in the case of trust in information sources, which suggests that acquisition of information through private activities is more important than trust in the government, which is a public agent managing public affairs to protect citizens’ interests.

Third, government accountability has a positive correlation with intention and action. The coefficient value was relatively small compared with other government variables. However, the correlation coefficient value of behavior was higher than that of intention. This indicates that belief in government accountability may be an important variable in promoting behavior rather than intention.

Fourth, policy satisfaction is positively correlated with both intention and behavior. It is noticeable that policy satisfaction had a higher correlation with behavior rather than with intention. This suggests that government satisfaction plays an important role in encouraging action, similar to knowledge and government accountability.

Finally, policy awareness shows a positive correlation with intention and action. Awareness had a higher correlation value with response intention than with behavior.

The last line is the correlation between the gap, which is the value of the action minus the intention, and other variables. As knowledge and government satisfaction increased, the gap widened, whereas when benefits and government trust increased, the gap decreased.

When looking at the relationship of intention and action with the above 10 variables, it can be seen that intention had the strongest correlation with government competence, which was followed by knowledge > policy awareness > negative emotion > policy satisfaction > trust in private information sources > perceived benefit > trust in government > perceived risk > government accountability. In the case of action, the largest correlation coefficient value was for knowledge, followed by government competence > policy awareness > policy satisfaction > negative emotions > trust in private information sources > trust in government > perceived risk > perceived benefit > government accountability. Thus, government competence and knowledge play a decisive role in shaping intentions and actions.

Table 3 shows that, overall, the variables had a higher correlation with intention than with action. However, knowledge, government accountability, and government satisfaction showed higher correlations with behavior than with intention. This suggests that these three variables may have a greater influence on action than on intention.

Also, there was some degree of difference in the coefficient values between intention and action. However, the difference between the two coefficient values was very small, such as for government competence. Hence, government competence could be the variable that can reduce intention–action inconsistency.

### 4.3. Regression Analysis

Table 4 shows the results of the regression analysis performed to identify the determinants of response intention and action against PM. For the calculation of dependent or independent variables in risk perception and government factors, the mean value was used when there were multiple measurement items. To check for multicollinearity, we calculated the tolerance and variance inflation factor (VIF). There is no multicollinearity when the VIF approaches 1, but there is multicollinearity if it is 10 or more. The figures in Table 5 confirm that there was no multicollinearity. In the case of tolerance, there is no multicollinearity when it is close to 1, and multicollinearity occurs when it is 0.1 or less. Since the tolerance value appeared to be from 0.201 to 0.917, we concluded that there was no multicollinearity in the model.

The column for Model 1 shows that the intention of taking action against PM is stronger in women and older people than in men and younger people. Since women and the elderly are more sensitive to external risks than men and younger people, the former displays a higher intention to respond than the latter. The regression coefficients are not significant in the case of income or residence, which suggests that there may be no difference in response intentions based on income group and residence. This appears because PM is harmful to everyone regardless of income or residence. Regarding health variables, it is expected that health vulnerabilities would make individuals more sensitive to the PM problem, resulting in stronger intention to respond. However, the numbers show that health status did not make a significant difference in intention or action, indicating that the PM issue may be a topic of interest to everyone regardless of health.

Among the variables in the psychometric paradigm, perceived benefits, negative emotions, trust, and knowledge affected response intention. The more benefits from solving PM respondents get, the more negative feelings, the higher the level of knowledge about PM, and the more active they are in responding. Interestingly, the effect of the perceived risk of fine dust was found to be insignificant. In general, the higher the perceived risk, the higher the level of response behavior. However, this hypothesis was not supported in this study. A possible reason is that since the perceived risk and the accompanying response intention were high for everybody, there was no clear linear relation in variance.

The variable with the greatest explanatory power was negative emotion, followed by knowledge, trust in private information sources, and perceived benefits. These results suggested that an emotional approach is necessary to induce public action for resolving the fine dust problem.

Among government variables, the higher the government competence, policy satisfaction, and policy awareness, the more action is taken against PM. Hence, changes and efforts are needed to invest in human capital and other factors to improve the government’s ability to mitigate PM-related problems. To increase policy satisfaction, it is necessary to design detailed policies that aim to enhance individual efficacy. Moreover, public communication to increase public awareness about PM policies is essential.

Table 4 shows that trust in private information sources in the risk perception paradigm had significant impact, while trust in government had insignificant impact. Thus, trust in private sources of information in the private domain had a stronger influence on intention than trust in the government in the public domain. These results show that since fine dust occurs every day in daily life, information sources in the private sector can play a large role. Government accountability was not statistically significant. It seems that because accountability has the attributes of norms that the government should have, it has a weaker influence compared with the actual competence, which is effectively working in the real world.

Based on the standardized regression coefficient value, the variable that showed the highest explanatory power among the government factors was government competency, followed by policy satisfaction and awareness. Policy satisfaction and awareness mainly concerned citizens who are policy recipients, whereas policy competence refers to the government as the provider of policy services related to PM. Thus, when designing and implementing policies related to PM, the primary focus must be on strengthening the government’s capabilities rather than enhancing policy satisfaction and policy awareness.

Model 1 had a high explanatory power of 45.5% and was statistically significant as well. The variables in this model showing the highest explanatory power were government competency, followed by negative emotions, knowledge, perceived benefits, policy awareness, and policy satisfaction. Thus, to strengthen response intention, it takes into account the perceptions of citizens, who are the main recipients of PM policy, as well as objective attributes related to the government, such as government competence, policy awareness, and policy satisfaction.

Model 2 shows that as the age increased, action against PM increased. For women, the coefficient was statistically significant for intention but not for action. The other four variables were not statistically significant, suggesting that response intention and action against PM are not significantly affected by education, income, health level, or residential area.

In the risk perception paradigm, negative emotions, trust in private information sources, and knowledge had a positive effect on response action, with knowledge having the greatest influence. In the case of intention against PM, the standardized regression coefficient values of negative emotion and knowledge were similar, but in the case of response action, that of knowledge was much larger than that of negative emotion. These results indicate that knowledge diffusion related to PM is necessary to induce action. It is interesting to note that the perceived benefit is related to the intention to respond to fine dust but not to the behavior. These results suggest that utilitarian incentives have some limitations in inducing behavior.

Among government variables, government competence, government accountability, policy satisfaction, and policy awareness had a positive effect on response action. The variable with the greatest influence was government competence, followed by policy satisfaction, government accountability, and policy awareness. Notably, government accountability, which was not significant for intention, was significant for action. Moreover, policy awareness had less influence on action than on intention. Thus, the role of government and policy variables should be differentiated between intention and action.

When comparing Models 1 and 2, the first difference is that when looking at variables such as gender, educational background, and perceived benefits that were significant in Model 1, they were not significant in Model 2. On the other hand, government accountability, which was not significant in Model 1, was significant in Model 2. Age, negative emotions, knowledge, government competency, and government satisfaction had a significant impact in both models. Thus, while considering common variables to induce response intention and behavior, it is important to consider the variables that bring out the difference in intention and behavior such as gender, educational level, perceived benefits, and government accountability to induce intention or behavior.

Model 2′s total explanatory power was 45.3%. For intention, the variable with the strongest influence was government competency, followed by negative emotion, knowledge, perceived benefit, policy awareness, and policy satisfaction. For action, the order, beginning with the strongest influence, was as follows: knowledge, government competence, government satisfaction, negative emotion, government accountability, and policy awareness. On the whole, the risk perception paradigm variables appeared prominently under intention, whereas government or policy variables appeared under action. Hence, the emphasis and attention in designing the response action should be different for intentions and actions. Additionally, since variables belonging to the risk perception paradigm and government factors simultaneously affect intention and action, the PM policy should consider the policy beneficiary’s perception structure as well as the policy itself.

### 4.4. Logistic Regression

To execute the logistic regression, we divided the respondents into four groups. Based on cluster analysis about intention and action variables, the respondents were divided into four groups: a high group and low group in intention and a high group and low group in action. The final distribution of respondents is shown in Table 5 below.

Looking at the consistent groups, 24.8% of the total respondents (124 out of 500) were categorized under the low-intention low-action group, and 24.2% (*N* = 121) were in the high-intention high-action group. On the other hand, the inconsistent groups consisted of the low-intention high-action group (30.2% (*N* = 151)) and the high-intention low-action group (20.8 % (*N* = 104)). It is evident that most respondents (69.6%) tried to maintain consistency in their intentions and actions.

We executed logistic regression to analyze the factors influencing (in)consistency (i.e., the membership of the four groups). As for the predicted dummy variable, the focus group was treated as one, and the other three groups were set to zero as the reference group. The result is shown in Table 6.

In Model 3, the focused group refers to those with low intention and low action against PM, and the reference group includes the remaining three groups. Women were more likely to not belong to this group. The higher the respondent’s age, the more likely he or she was to not belong to the focused group because as age increases, intention or action are likely to appear because older people are sensitive to the hazards of PM.

Among variables of perception factor, perceived risk increased the likelihood of belonging to this group. Similarly, more negative emotions and a high level of knowledge reduced the probability of belonging to this group. Thus, negative emotions and knowledge are influential factors that weaken intentions and action. In addition, on the other hand, belief in government competence led to a low possibility of belonging to the low-intention low-action group. Thus, government capability also possibly strengthens response intention and action.

In Model 6, the focus group includes respondents with high intention and high action. Among the variables in the risk perception paradigm, the higher the level of negative emotions, trust, and knowledge, the more likely one was to belong to this group. Similarly, higher government competence and policy satisfaction increased the probability of belonging to this group.

Since Model 3 and Model 6 are opposite groups, the significant variables were expected to show similar patterns, even if the direction of impact was reversed. However, the results did not confirm this assumption. In both models, negative emotions, knowledge, and government competence were significant, but trust in private information sources and policy satisfaction were significant only in Model 6. Thus, the determinants of belonging to the two groups may be different. For example, if trust in private information sources is high, the respondent shows a higher probability of belonging to a high-intention high-action group, but it does not mean that he or she belongs to the low-intention low-action group.

Model 4 is a case where actual behavior occurs, although the response intention is low. It is the most difficult group to interpret from the perspective of rational thinking in that people act without intention. The most likely explanation is that they find themselves in a situation wherein failure to take action against PM would soon endanger their health or life. Therefore, even though they had not thought about it or wanted to do it, they ended up, perhaps “unwillingly”, taking action. Variables that significantly affected the chances of belonging to this group are perceived risk, benefits, knowledge, trust in government, and government accountability. One is less likely to belong to this group when there is high perceived risk and perceived benefit. Knowledge increases the likelihood of belonging to this group. Thus, perceived risks and benefits can destroy willingness to act, whereas high knowledge can convince one to act despite reluctance. Reinforcement of trust and accountability in the government undermines membership in this group. When individuals learn about how PM can harm them, they are likely motivated to take action even if they had not initially intended to.

Model 5 is about a group that has high intention but does not actually act. In the case of women, the higher the age, the higher the probability of belonging to this group, whereas the higher the income, the lower the probability of belonging to this group. As the perceived benefits increase, the likelihood of belonging to this group increases. But as trust in government increases, the likelihood of belonging to this group decreases. It is highly likely that increasing the perceived benefits will not induce behavior in those with intentions. On the other hand, if knowledge increases, it is unlikely that they will not be able to induce action from those with high intentions. However, trust in government plays the opposite role.

When looking at Models 3, 4, and 5 together, the variable that affected the possibility of belonging to all four groups was knowledge. Knowledge lowered the possibility of belonging to groups in Models 4 and 5, whereas it increased the possibility of belonging to groups in Models 5 and 6. The difference between Models 3 and 5 and 4 and 6 is whether or not the respondents act. Therefore, when knowledge is high, it induces behavior rather than non-action, regardless of whether the intention is high or low.

Variables that were significant in two models included female, age, perceived risk, benefits, negative emotion, government competence, and trust in government. However, the roles of these seven variables were different. Emotion and government competence helped promote intention–action consistency, whereas perceived benefits led to inconsistent behavior. Moreover, perceived risk and policy satisfaction affected consistent and inconsistent behavior simultaneously.

Variables that affected belonging to only one group were income, living in a large city, trust in private information sources, and policy satisfaction. If all those variables increased, there was a high possibility of belonging to the high-intention high-action group.

The above results show that the possibility of belonging to the four groups varied from model to model. Knowledge affected both consistent and inconsistent behavior at the same time. Negative emotions and government competence affected consistent behavior, whereas perceived benefit and trust in the government influenced only inconsistent behavior. Trust in a source and policy satisfaction were influential only for the high-intention high-action group, whereas government accountability influenced only the low-intention high-action group.

## 5. Discussion and Theory or Policy Implications

In terms of theory, many studies have been conducted on the relationship between intention and behavior. In particular, a lot of research mainly used the theory of reasoned action (TRA) and the theory of planned behavior (TPB) to explain intention and behavior [60,61,62,63,64,65]. In several studies, various factors were analyzed by focusing on the gap between intention and behavior. For example, Tonglet et al. [66] showed that pro-environmental attitudes are the main factor that leads to participation in waste recycling behavior. In addition, Garces et al. [67] showed that individuals’ perceptions and knowledge of the environment are factors that lead to pro-environmental behavior. However, these studies were relatively less interested in the gap between intention and behavior itself [68]. Andorfer and Liebe [69] also pointed out these issues. There was a lack of research on the relationship between behavioral intention and behavior. In addition, existing research lacks a holistic framework investigating the gap between intention and action. This study is meaningful in that it helps fill those research gaps.

In terms of practical implication, the above analysis has implications for how governments can develop PM policies that induce response actions against PM. In terms of closing the intention to action, governments should consider the policy implications of this study. The first thing to consider while making efforts to encourage citizens to take action against PM is that policymakers acknowledge that there is a gap between intention and behavior. This study shows that intentions do not necessarily lead to actions. Therefore, it is necessary to make policy efforts to convert intentions into tangible action. By analyzing the gap between intention and action, we confirmed that there exist not only consistent groups but also inconsistent groups. Therefore, the policy object can be specifically categorized by considering the composition between consistent groups and inconsistency groups. The government should manage groups that show inconsistencies rather than groups that show consistency. This study presents a useful insight for policy intervention by showing factors that play an important role in cases where intention does not lead to action. In particular, the government needs to pay attention to the group that has intentions but does not take action. For these groups, active management by using variables such as perceived benefit and policy satisfaction is required.

Second, when comparing the two models in regression analysis, not only the variables belonging to the risk perception paradigm but also the variables related to government factors were found to be significant. If the former is mainly related to the subjective perception of policy recipients, then the latter is related to the objective qualities of the government as a policy provider. This means that in order to induce a response of action against PM, policymakers should increase their understanding of the determinant structure of inconsistency in intention and action.

Third, it is necessary to manage variables that simultaneously affect intention and behavior. In this study, negative emotions, trust in an information source, knowledge, government accountability and competence, and policy satisfaction or awareness affect intention and behavior. The negative image of the PM should be emphasized when promoting or communicating with the public. In addition, education and training should be strengthened in order to increase the knowledge level related to PM. In order to increase the reliability of information, the accuracy, quality, and timeliness of information must be considered. Moreover, in order to strengthen the government’s capacity and accountability, it is necessary to not only reinforce the physical infrastructure that can analyze PM but also strengthen manpower competence by increasing education and imbuing the public mind. In addition, it is necessary to evaluate citizens’ satisfaction with PM-related government policies and the management thereof. Our research suggests that acceptance is important when implementing a policy. Therefore, it is necessary to consider these aspects when implementing policies.

Fourth, the most ideal group in PM policy is the high-intention high-action group. However, the logistic analysis showed that in addition to this ideal group, there are low-intention low-action, high-intention low-action, and low-intention high-action groups. In particular, the problematic groups are the low-action groups (low-intention low-action group and high-intention low-action group). In order to shrink those groups, policy design must consider five variables: perceived risk, benefits, negative emotions, knowledge, and government competence. In addition, it is necessary to understand the variables that induce consistency and inconsistency between intention and action. Perceived benefits and trust in government induce inconsistency, whereas negative emotions and government competence induce consistency. Knowledge creates both consistency and inconsistency at the same time. In order to reduce inconsistency and increase consistency, it is necessary to design a policy that takes these variables into consideration.

Finally, in order to induce action against PM, it is necessary for the public to have a response intention before action. In this study, the variables that only affected intention were the credibility of information sources and policy awareness. PM-related information obtained through informal channels may have the potential to cause mistrust related to PMs. Therefore, the government needs to provide reliable information to the general public. In addition, a more active public relations strategy is needed to increase the number of people who are aware of public policies about PM.

## 6. Conclusions

The purpose of this study was to analyze comparatively how the predictors affect response intention and action against PM. In particular, this study focused on (in)consistency between intention and action. To identify the factors influencing consistency, we divided the respondents into four groups according to their response levels of intention and action (high and low). Next, we analyzed how risk perception and government factors affect the likelihood of belonging to each group. The main findings of this study are as follows.

First, there was a gap between intention and action. To compare two variables, we calculated the mean of the measurements. The respondents scored 3.344 out of 5 for their intentions to take preventive action against PM and 2.982 out of 5 for their actual action against PM. Thus, generally, intention does not lead to action against PM. This result was not unexpected. Since it takes time and money to implement an intended action, intention will generally not be implemented. However, since the two variables were measured subjectively, there is a limit to this comparison.

Second, regression analysis showed commonalities and differences in the influence of variables affecting response intention (Model 1) and action (Model 2). Age, negative emotions, trust in private information sources, knowledge, government competence, satisfaction with government, and policy awareness had a common influence in determining intentions and actions. Gender, educational level, and perceived benefits were significant in determining intentions but not action. Government accountability was not significant for intention but was for action. Moreover, another difference between the two models was the explanatory power of the independent variables. In the case of intention, this was in the order of government competence > negative emotions > knowledge > perceived benefits > policy awareness > policy satisfaction, whereas for action, it appeared in the order of knowledge > government competency > policy satisfaction > negative emotions > age. Risk perception paradigm variables are significant for intention, and government or policy variables are significant in influencing behavior.

Third, through logistic analysis, we examined the likelihood of belonging to the four groups according to the classification of intention and action. Negative emotions and government competence affected the likelihood of affiliation with a consistent group (low-intention low-action or high-intention high-action). On the other hand, perceived benefits and trust in government affected affiliation with the inconsistent groups (high-intention low-action and low-intention high-action). Perceived risk and knowledge are variables that induce both consistent and inconsistent responses. Trust in private information sources and policy satisfaction played a role in inducing high intention and high action, whereas government accountability induced low intention and high action.

## 7. Limitations

This study analyzed consistency and inconsistency in PM response intention and action by focusing on the risk perception paradigm and government factors. The first limitation of this study is that the sample was small, being only 500 respondents. Hence, national representativeness was not adequately achieved. Second, in logistic analysis, it is possible to analyze various groups depending on how the reference group is set up, but in this study, only four groups were assumed. Third, there is a limitation in setting the predictors that affect response intention and behavior. We did not include social, political, or economic factors, which may potentially be as important as perceptual and government factors.

## Figures and Tables

**Table 1:** Percent of population and sample.

	Gender		Age				
	Male	Female	20s	30s	40s	50s	Over 60s
Population	21,430,423	21,694,995	7,031,016	7,106,801	8,232,362	8,582,699	12,172,540
%	49.7	50.31	16.3	16.48	19.09	19.9	28.23
Sample	246	254	83	76	92	99	150
%	49.2	50.8	16.6	15.2	18.4	19.8	30

**Table 2:** Concepts, measurement items, and the scale’s reliability.

	Factors/Variables		Measurement Items	Cronbach’s α	Average (SD)
Control variables	Subjective health conditions		I am healthy.	0.851	3.247 (0.794)
			I am healthier than other people.		
	Residential area		Where do you live? (1) Large city, (2) Small- or medium-sized city, (3) Rural area, or (4) Other	-	1.490 (0.602)
Independent variable	Risk perception factors	Perceived risk	A disease caused by PM has very serious consequences.	0.740	3.634 (0.716)
			A disease caused by PM will greatly interfere with my life.		
		Perceived benefit	If the PM problem is resolved, it will be a great benefit to our society.	0.697	3.866 (0.700)
			If PM is resolved, our society will develop greatly.		
		Negative emotion	PM gives me fear.	0.800	3.382 (0.820)
			PM makes me nervous.		
		Trust (private)	How much do you trust the sources below?	0.619	3.084 (0.734)
			(1) Online media (Internet newspapers, portal news, etc.) (2) Internet information sources (such as personal blogs, SNS, cafes, and communities).		
		Knowledge	I know more about PM than other people.	0.820	2.940 (0.826)
			I can explain to others the policies or issues related to PM.		
	Government factors	Government competence	The government seems to be trying to communicate with the public about the PM problem.	0.611	3.165 (0.783)
			The government has the capacity to control PM emissions.		
		Trust in government	How much do you trust the sources below?	0.700	3.497 (0.831)
			Government, (2) Public environment-related agencies (Ministry of Environment, National Institute of Environmental Sciences, Korea Environment Corporation, Fine Dust Information Center, and Korea Meteorological Administration)		
		Government accountability	The government is more responsible than individuals for PM generation.	0.785	3.546 (0.890)
			The government should be responsible for resolving PM rather than individuals.		
		Policy satisfaction	I am pleased with the government’s PM reduction policy.	0.824	2.877 (0.811)
			The government tries to inform the people about the PM policy and listens to the opinions of the people.		
		Policy awareness	Fine dust season control system	0.781	3.144 (0.708)
			A two-part public vehicle system		
			Expansion of LPG cars and eco-friendly cars		
			Restriction on operation of automobile emissions of Class 5		
			Support for installation of air purification facilities in public transportation vehicles		
			Dissemination of health masks to sensitive and vulnerable people		
Dependent variable	Intention to respond to PM		I will use public transportation rather than personal vehicles to reduce PM.	0.796	3.344 (0.644)
			I am willing to participate in a government petition to reduce PM.		
			I am willing to practice energy conservation to reduce PM.		
			I am willing to donate to environmental organizations that are engaged in related activities to reduce PM.		
			I am willing to pay the necessary costs for government-level projects to reduce PM.		
			I am willing to participate in a PM program or project promoted by the government.		
	Action to respond to PM		I use public transportation rather than personal vehicles to reduce PM.	0.810	2.982 (0.718)
			I am participating in a government petition to reduce PM.		
			I practice energy conservation to reduce PM.		
			I have experience in donating to environmental organizations that are engaged in activities related to reducing PM.		
			I have made financial contributions to government-level projects to reduce PM.		
			I have experience or participate in PM-related programs or projects promoted by the government.		

**Table 3:** Correlation analysis results.

Factors	1	2	3	4	5	6	7	8	9	10	11	12
1. Perceived risk	1											
2. Perceived benefit	0.419 ***	1										
3. Negative emotion	0.453 ***	0.287 ***	1									
4. Trust in private information source	0.196 ***	0.158 ***	0.189 ***	1								
5. Knowledge	0.171 ***	0.085	0.364 ***	0.243 ***	1							
6. Government competence	0.124 **	0.145 **	0.229 ***	0.314 ***	0.359 ***	1						
7. Trust in government	0.180 ***	0.273 ***	0.109 *	0.354 ***	0.123 **	0.308 ***	1					
8. Government accountability	0.202 ***	0.214 ***	0.218 ***	0.135 **	0.074	−0.070	−0.034	1				
9. Policy satisfaction	−0.010	0.025	0.067	0.256 ***	0.278 ***	0.551 ***	0.316 ***	−0.223 ***	1			
10. Policy awareness	0.224 ***	0.234 ***	0.204 ***	0.260 ***	0.371 ***	0.382 ***	0.367 ***	0.079	0.292 ***	1		
11. Intention	0.264 ***	0.311 ***	0.403 ***	0.325 ***	0.435 ***	0.483 ***	0.279 ***	0.088 *	0.351 ***	0.422 ***	1	
12. Action	0.168 ***	0.115 *	0.346 ***	0.288 ***	0.540 ***	0.481 ***	0.170 ***	0.108 *	0.383 ***	0.365 ***	0.682 ***	1
13. Action-intention gap	−0.091 **	−0.215 ***	−0.020	−0.005	0.197 ***	0.063	−0.104 **	0.038	0.089 **	−0.018	−0.282 ***	0.509 ***

Note: * *p* < 0.05. ** *p* < 0.01. *** *p* < 0.001.

**Table 4:** Regression analysis.

		Model 1: Intention					Model 2: Action				
		B	SE	β	Tolerance	VIF	B	SE	β	Tolerance	VIF
(Constant)		−0.077	0.26				0.077	0.29			
Female		0.090 *	0.045	0.07	0.917	1.09	0.035	0.05	0.024	0.917	1.09
Age		0.005 **	0.002	0.105	0.841	1.189	0.006 **	0.002	0.119	0.841	1.189
Above a college degree		0.1	0.056	0.065	0.836	1.197	0.034	0.063	0.02	0.836	1.197
Income		0.031	0.031	0.036	0.905	1.105	−0.012	0.034	−0.013	0.905	1.105
Health state		−0.001	0.03	−0.001	0.826	1.21	−0.006	0.034	−0.006	0.826	1.21
Residential area	Metropolis	0.141	0.099	0.108	0.195	5.125	0.112	0.111	0.078	0.195	5.125
	Small and medium size	0.044	0.1	0.033	0.201	4.984	0.004	0.112	0.003	0.201	4.984
Risk perception factors	Perceived risk	−0.002	0.037	−0.002	0.665	1.504	−0.009	0.041	−0.009	0.665	1.504
	Perceived benefit	0.129 ***	0.036	0.14	0.738	1.355	−0.034	0.04	−0.033	0.738	1.355
	Negative emotion	0.136 ***	0.032	0.174	0.666	1.501	0.110 **	0.036	0.125	0.666	1.501
	Trust in info. Source	0.074 *	0.034	0.084	0.761	1.315	0.065	0.038	0.067	0.761	1.315
	Knowledge	0.135 ***	0.032	0.173	0.664	1.507	0.283 ***	0.036	0.325	0.664	1.507
Government factors	Gov. competence	0.174 ***	0.036	0.212	0.585	1.711	0.196 ***	0.04	0.214	0.585	1.711
	Trust in government	0.014	0.031	0.018	0.692	1.446	−0.037	0.035	−0.042	0.692	1.446
	Gov. accountability	0.005	0.027	0.007	0.8	1.25	0.066 *	0.03	0.081	0.8	1.25
	Policy satisfaction	0.088 *	0.034	0.11	0.603	1.659	0.139 ***	0.038	0.158	0.603	1.659
	Policy awareness	0.110 **	0.037	0.121	0.681	1.469	0.075	0.041	0.074	0.681	1.469
N		500					500				
R2		0.455					0.453				
adj. R2		0.436					0.433				
F(p)		23.664 ***					23.447 ***				

Note: For the reference group, gender = male, education background = below a college degree, and residential area = rural. * *p* < 0.05. ** *p* < 0.01. *** *p* < 0.001.

**Table 5:** Classification of groups.

*N* = 500 (100%)		
Grouping	Low Action	High Action
Low Intention	**Low-Intention Low-Action Group** Respondents with low levels of response intention and low levels of response action *N* = 124 (24.8%)	**Low-Intention High-Action Group** Respondents with low levels of response intention but high levels of response action *N* = 151 (30.2%)
High Intention	**High-Intention Low-Action Group** Respondents with high levels of response intention but low levels of response action *N* = 104 (20.8%)	**High-Intention High-Action Group** Respondents with high levels of response intention and high levels of response action *N* = 121 (24.2%)

**Table 6:** Binomial logistic regression.

		Group with Low Intentions						Group with High Intentions					
		Model 3: Low-Intention Low-Action			Model 4: Low-Intention High-Action			Model 5: High-Intention Low-Action			Model 6: High-Intention High-Action		
		B	SE	Exp(B)	B	SE	Exp(B)	B	SE	Exp(B)	B	SE	Exp(B)
(Constant)		9.107 ***	1.499	9020.997	2.417	1.321	11.213	−4.414 *	2.180	0.012	−16.898 ***	1.978	0.000
Female		−0.249	0.234	0.780	0.034	0.241	1.034	0.489	0.341	1.630	0.020	0.254	1.020
Age		−0.026 **	0.008	0.974	0.013	0.009	1.013	0.010	0.012	1.010	0.021 *	0.010	1.021
Above a college degree		−0.150	0.289	0.861	−0.103	0.293	0.902	0.787	0.492	2.196	0.065	0.321	1.067
Income		0.063	0.156	1.065	−0.117	0.156	0.890	−0.282	0.221	0.754	0.227	0.181	1.255
Health state		−0.200	0.156	0.819	0.141	0.165	1.151	0.219	0.210	1.245	−0.153	0.180	0.858
Residential area	Metropolis	−0.053	0.521	0.949	−0.024	0.503	0.977	1.411	1.064	4.101	−0.069	0.594	0.933
	Small- and medium-sized	0.203	0.524	1.226	0.032	0.508	1.033	0.943	1.080	2.568	−0.176	0.600	0.839
Risk perception factors	Perceived risk	0.284	0.200	1.329	−0.224	0.187	0.799	−0.364	0.294	0.695	−0.066	0.217	0.936
	Perceived benefit	−0.003	0.190	0.997	−0.549 **	0.188	0.577	0.853 **	0.297	2.346	0.224	0.213	1.251
	Negative emotion	−0.543 **	0.169	0.581	−0.243	0.170	0.784	0.318	0.251	1.374	0.747 ***	0.201	2.111
	Trust in private information source	−0.250	0.180	0.779	−0.159	0.181	0.853	−0.050	0.241	0.952	0.543 **	0.201	1.721
	Knowledge	−0.747 ***	0.171	0.474	0.423 *	0.181	1.527	−0.413	0.224	0.661	0.729 ***	0.204	2.072
Government factors	Competence	−0.665 ***	0.190	0.514	−0.033	0.199	0.968	0.184	0.251	1.202	0.737 **	0.215	2.089
	Trust in government	0.016	0.164	1.016	−0.150	0.165	0.861	0.166	0.228	1.181	−0.021	0.182	0.979
	Accountability	−0.210	0.146	0.811	−0.013	0.149	0.987	−0.249	0.198	0.780	0.417 *	0.165	1.518
	Policy satisfaction	−0.302	0.188	0.739	0.043	0.190	1.044	−0.481 *	0.241	0.618	0.647 **	0.203	1.911
	Policy awareness	−0.256	0.193	0.775	−0.212	0.198	0.809	−0.062	0.270	0.940	0.447 *	0.218	1.564
N		500			500			500			500		
X^2^		163.105 ***			40.303 **			32.774 *			225.432 ***		
-2LL		489.154			468.305			287.892			414.268		
Cox and Snell		0.278			0.077			0.063			0.363		
Nagelkerke R^2^		0.382			0.121			0.134			0.503		

Note: For the reference group, gender = male, education background = below a college degree, and residential area = rural. * *p* < 0.05. ** *p* < 0.01. *** *p* < 0.001.

## Data Availability

The data presented in this study are available on request from the corresponding author. The data are not publicly available due to regulations and guidelines for the data.

## Appendix A

**Table A1:** Factor Analysis for Risk Perception Factors.

Variables	Factor Loading					h^2^	Cronbach’s α
	Knowledge	Negative Emotion	Perceived Benefit	Perceived Risk	Trust (Private)		
Knowledge1	0.838	0.193	−0.024	0.022	0.144	0.851	0.82
Knowledge2	0.779	0.143	0.054	0.085	0.112	0.844	
N_emotion1	0.144	0.765	0.139	0.234	0.118	0.835	0.8
N_emotion2	0.238	0.739	0.121	0.193	0.049	0.831	
P_Benefit1	0.102	0.147	0.707	0.163	0.141	0.781	0.697
P_Benefit2	−0.065	0.082	0.702	0.223	0.008	0.784	
P_Risk1	0.057	0.155	0.253	0.701	0.082	0.824	0.74
P_Risk2	0.068	0.301	0.202	0.681	0.111	0.778	
Trust1	0.035	0.055	0.125	0.039	0.691	0.770	0.619
Trust2	0.187	0.071	−0.003	0.105	0.627	0.726	
eigenvalue	1.740	1.686	1.572	1.562	1.464	-	-
% variance	17.399	16.863	15.720	15.622	14.639	-	-
% accum.	17.399	34.262	49.982	65.604	80.244	-	-

KMO = 0.718. Bartlett’s X^2^ = 1516.036 (df = 45, *p* = 0.000).

## Appendix B

**Table A2:** Factor Analysis for Government Factors.

Variables	Factor Loading					h^2^	Cronbach’s α
	Policy Awareness	Policy Satisfaction	Trust in Government	Accountability	Competence		
P_Awareness3	0.735	0.073	0.245	0.047	−0.082	0.614	0.781
P_Awareness5	0.734	0.257	−0.033	0.067	−0.001	0.610	
P_Awareness4	0.728	−0.051	0.188	0.082	0.043	0.576	
P_Awareness1	0.630	0.160	0.093	0.024	0.246	0.493	
P_Awareness6	0.601	0.277	−0.076	−0.089	0.212	0.496	
P_Awareness2	0.547	−0.273	0.484	0.001	0.163	0.636	
P_Satisfaction2	0.163	0.848	0.077	−0.087	0.139	0.779	0.824
P_Satisfaction1	0.103	0.835	0.164	−0.144	0.113	0.769	
G_Trust2	0.137	0.061	0.863	0.042	0.063	0.773	0.7
G_Trust1	0.116	0.365	0.759	−0.056	0.044	0.728	
G_accountability2	0.077	−0.099	0.030	0.900	−0.040	0.829	0.785
G_accountability1	0.034	−0.125	−0.028	0.891	0.063	0.816	
G_competence1	0.238	0.570	0.109	−0.104	0.476	0.630	0.611
G_competence2	0.122	0.230	0.094	0.059	0.887	0.867	
eigenvalue	2.813	2.209	1.721	1.675	1.198	-	-
% variance	20.092	15.777	12.291	11.962	8.559	-	-
% accum.	20.092	35.869	48.16	60.122	68.681	-	-

KMO = 0.805. Bartlett’s X^2^ = 2173.655 (df = 91, *p* = 0.000).
